# A complete *Candidatus walczuchella monophlebidarum* genome assembled from citrus leaf metagenomic sequences

**DOI:** 10.1128/mra.00620-26

**Published:** 2026-07-10

**Authors:** Günther Koraimann, Nina Hölzl, Michael Koller, Gernot Zarfel, Fritz Treiber

**Affiliations:** 1 Institute of Molecular Biosciences, University of Graz 27267, Graz, Austria; 2 Studiengang Biomedizinische Analytik, Fachhochschule JOANNEUM, Graz, Austria; 3 Diagnostik- und Forschungsinstitut für Hygiene, Mikrobiologie und Umweltmedizin, Medizinische Universität Graz 31475, Graz, Austria; Rochester Institute of Technology, Rochester, New York, USA

**Keywords:** insect endosymbiont, metagenome, citrus, giant-scale insect, genomes

## Abstract

We present the complete *de novo* assembly of a *Candidatus Walczuchella monophlebidarum* genome (286,606 bp), a flavobacterial endosymbiont of the giant-scale insect *Icerya purchasi*. The genome was assembled from metagenomic short read Illumina sequences obtained from DNA of citrus leaves collected in Carinthia, Austria in November 2024.

## ANNOUNCEMENT

*Candidatus Walczuchella monophlebidarum* is a flavobacterial endosymbiont of giant-scale insects that feed on plant leaves. *Walczuchella monophlebidarum* bacteria evolved in their respective insect host and have a highly reduced genome with a size of approximately 300 kbp and a high frequency of pseudogenization (1).

We discovered a complete *Walczuchella* genome in a metagenomic analysis of DNA extracted from upper and lower sides of six citrus leaves from a citrus horticulture in Carinthia, Austria (N 46.56282, E 13.91066). Leaf sampling was done using sterile equipment; bags containing leaves were stored at 4°C until further processing. DNA extraction was performed by using the DNeasy PowerWater Kit (QIAGEN). Leaves were treated similar to filters used in the kit according to the manufacturer's instructions. DNA concentrations were photometrically determined by NanoDrop. DNA samples were then processed through the “INVIEW Metagenome” pipeline at the next-generation sequencing facility of Eurofins Genomics (Germany). The Illumina-compatible library was prepared using the unique dual-indexing technique and sequenced on a NovaSeq 6000 (PE 150). For data on raw sequence reads, see Table 1.

**TABLE 1:** Citrus leaf sources and total raw reads for each sample are given

Sample^*a*^	Description	Total raw reads
1AOS	*Citrus paradisi* Royal upper side of the leaf	49.88 M^*b*^
1AUS	*Citrus paradisi* Royal underside of the leaf	50.57 M
2AOS	*Citrus aurantifolia* Messicana upper side of the leaf	45.19 M
2AUS	*Citrus aurantifolia* Messicana underside of the leaf	44.82 M
3AOS	*Citrus bergamia* Fantastico Royal upper side of the leaf	45.7 M
3AUS	*Citrus bergamia* Fantastico underside of the leaf	52.65 M
4AOS	*Citrus aurantifolia* della Corsica upper side of the leaf	50.82 M
4AUS	*Citrus aurantifolia* della Corsica underside of the leaf	50.36 M
5AOS	*Citrus paradisi* Star Ruby upper side of the leaf	48.96 M
5AUS	*Citrus paradisi* Star Ruby underside of the leaf	50.17 M
6AOS	*Citrus limon* salicifolia upper side of the leaf	23.99 M
6AUS	*Citrus limon* salicifolia underside of the leaf	50.5 M

^*a*^ Short name for each sample.

^*b*^ “M” indicates million.

Paired-end reads were quality checked and trimmed using sickle 1.33 (2). Contigs from a total of 12 samples were assembled with megahit 1.2.9 (3) on the GalaxyEU webserver (4). Next, 248,039 assembled contigs with a minimal sequence length of 1,000 bp were downloaded from the server as a multi-fasta file and classified with kraken2 2.0.8-beta (5) using a k2_pluspfp database limited to 16 GB (k2_pluspfp_16gb_20241228). Contig sequences were then filtered to contain bacterial and fungal sequences only using Krakentools 1.2 (6). The remaining 1,274 contigs (26,981,927 nucleotides) were further analyzed in the Anvi'o 8 environment (7).

Contigs were grouped based on sequence composition and differential coverage across the 12 samples, revealing the presence of genome sequences from one fungus (genus: *Cladosporium*) and two endosymbiotic bacteria (*Walczuchella* and *Wolbachia*). A first taxonomic classification using GTDB R214.1 (8) yielded *Wolbachia pipientis* and *Walczuchella monophlebidarum* as being the only bacterial species detected. Protein BLAST (blastp 2.17.0+) searches at the National Center for Biotechnology Information (NCBI) webserver (accessed October 2025) with selected protein sequences from the *Walczuchella* contig (286,759 bp) revealed hits with 100% amino acid sequence identity only to *Walczuchella* from *Icerya purchasi* (NCBI BioProject number PRJEB75090).

The *Walczuchella* contig was mainly derived from one sample (2AOS, *Citrus aurantifolia* Messicana upper side of the leaf) with a mean coverage of 75. Repetitive sequences (153 bp) at the sequence ends of this contig were removed to generate a circular chromosome of 286,606 nucleotides, representing the complete genome.

The guanine-cytosine content was found to be 32.51%. A total of 264 protein-coding genes were detected with prodigal 2.6.3 (9), and genes were annotated using PFAM38 (10), Kyoto Encyclopedia of Genes and Genomes (11), and COG24 (12, 13) databases. Ribosomal RNAs (23S/16S—1/1) and 35 tRNAs were determined using *anvi-run-hmms* and *anvi-run-trnas*, respectively, based on tRNAScan-SE 2.0.12 (14) within Anvi'o 8 (7). Default parameters were used for all programs.

## Data Availability

Sequence data associated with this publication have been deposited to the European Nucleotide Archive (ENA) at EMBL-EBI under accession number PRJEB104488. The assembled genome is available under under accession number OZ464853.
